# A 10-year registry-based retrospective study of cutaneous Leishmaniasis in Mashhad, northeastern Iran: Demographic, clinical, and therapeutic profiles of 1537 cases

**DOI:** 10.1016/j.parepi.2026.e00478

**Published:** 2026-02-05

**Authors:** Vahid Mashayekhi-Goyonlo, Pouran Layegh, Zahra Ghasemi, Masomeh Hosseini-Nezhad, Ali Tajik

**Affiliations:** a Cutaneous Leishmaniasis Research Center, Mashhad University of Medical Sciences, Mashhad, Iran; b Student Research Committee, Faculty of Medicine, Mashhad University of Medical Sciences, Mashhad, Iran

**Keywords:** Cutaneous leishmaniasis, Epidemiology, Iran, Treatment outcomes, Retrospective study

## Abstract

**Background:**

Cutaneous leishmaniasis (CL) remains one of the most prevalent neglected tropical diseases, imposing a substantial health burden in many developing countries. Given the importance of epidemiological data in guiding public health responses, this retrospective registry-based study aimed to describe and compare the epidemiological, demographic, clinical, and therapeutic characteristics of CL among patients referred to the Cutaneous Leishmaniasis Research Centers in Mashhad, Northeastern Iran, between 2015 and 2024.

**Methods:**

This retrospective case-series included all patients (*n* = 2031) referred to the CL Research Center bases at *Imam* Reza Hospital, Ghaem Hospital, and Abo-Bargh Health Center (Mashhad, Iran) with suspected CL during 2015–2024.

**Results:**

Among 1537 confirmed CL cases, children under 10 years accounted for the largest proportion (34.9%). Male patients were slightly more frequent than females (52.3%). The majority had low educational levels (71.6%) and resided in peri-urban areas (59.4%). Almost all cases were localized (99.4%), with lesions predominantly on exposed areas such as the head and neck (47.0%) and upper extremities (38.6%). The most common clinical forms were papulonodular (50.4%), ulcerative (31.0%), and plaque-type lesion (13.7%). Intralesional antimony was the most frequent treatment (59.3%), and approximately 63% of treated patients achieved complete cure. Seasonally, case numbers peaked in autumn, declined in winter, and rose again in spring.

**Conclusion:**

CL remains a major public health concern in Mashhad, Iran. Despite encouraging cure rates, the true disease burden is likely underestimated. Demographic and climatic factors influence its distribution. Longitudinal and clinical studies are warranted to better understand treatment outcomes. Strengthened surveillance, public awareness, and coordinated control programs are crucial for sustainable disease management.

## 1 Introduction

Leishmaniasis comprises a group of vector-borne diseases caused by protozoan parasites of the Leishmania genus (Grifferty et al., 2023). Transmission occurs through the bite of infected phlebotomine sand flies, *Phlebotomus* species in the Old World and *Lutzomyia* species in the New World, depending on the geographical region (Shokri et al., 2017). The disease exhibits a wide clinical spectrum, ranging from self-healing cutaneous lesions to chronic mucocutaneous and potentially fatal visceral forms, largely influenced by parasite species and host immune response (Salam et al., 2014).

Among these, cutaneous leishmaniasis (CL) is the most prevalent yet least fatal form, and it remains endemic in many regions of the world (Reithinger et al., 2007). CL is endemic across many widely separated regions of the world. The classical form of Old World CL, often referred to as the “oriental sore,” has been known by numerous local names, such as *bouton de Crète, bouton d'orient, bouton d'Alep, Baghdad boil, Aleppo evil, bouton de Biskra,* and *Delhi boil*, in different parts of the Mediterranean basin, Middle East, India, Africa, and Asia (Zumla, 2010). According to the World Health Organization's (WHO) latest surveillance data on 2023, CL is endemic in over 55 countries, with the highest burdens reported in Pakistan, Brazil, Afghanistan, Algeria, Syria, and Peru (World Health Organization, 2024). It is estimated that 600,000 to 1 million new cases occur worldwide annually but only around 200,000 are reported to WHO (World Health Organization, 2023). Among the endemic countries, Iran has a long history of epidemiologic research on leishmaniases, dating back to 1941 (Azizi et al., 2016). The disease was documented in ancient Persian medical literature by several renowned Iranian scholars. Abū Bakr Muhammad ibn Zakariyyā al-Rāzī (854–925 CE) referred to the lesion as “Pashegezidegi,” meaning “mosquito's bite” in Persian. Later, Avicenna (Ibn Sina, 980–1037 CE) described a similar cutaneous condition in The Canon of Medicine (Azizi et al., 2016). Comparable descriptions of lesions consistent with CL also appear in other classical works, including Majma-ol-Javame by Alavi and Sharh-e-Asbab by Mollanafis (Azizi et al., 2016). The annual incidence of CL in Iran is estimated at 30.9 cases per 100,000 population, corresponding to roughly 20,000 new cases each year, underscoring its continued public health significance. (Badirzadeh et al., 2017; Norouzinezhad et al., 2016; Holakouie-Naieni et al., 2017). Two main forms are recognized: zoonotic cutaneous leishmaniasis (ZCL), caused by *Leishmania major*, and anthroponotic cutaneous leishmaniasis (ACL), caused by *Leishmania tropica* (Piroozi et al., 2019).

The distribution and persistence of CL within Iran are strongly influenced by ecological and socio-environmental factors, including urbanization, migration, and climate variability, which shape the habitats of sand-fly vectors and animal reservoirs (Ghatee et al., 2020; Karimi et al., 2021). Typically, *L. major* predominates in rural and arid foci, whereas *L. tropica* is more common in urban and peri-urban centers (Ghatee et al., 2020). Infections caused by L. *tropica* (ACL) are generally more challenging to diagnose and treat and tend to persist longer than those due to L. *major* (Klaus and Frankenburg, 1999; Dowlati, 1996).

Clinically, CL presents with diverse manifestations such as papular, nodular, ulcerative, plaque-type, and chronic lupoid forms, along with less common variants like sporotrichoid or erysipeloid lesions (Zumla, 2010; Kouki et al., 2022; Carvalho et al., 2017). Treatment options for CL include systemic and intralesional antimonial drugs, cryotherapy, thermotherapy, and topical formulations such as paromomycin-based ointments (Zumla, 2010). The choice of therapy depends on lesion type, number, location, and resource availability, as well as species-specific response patterns. Patterns of clinical presentation and therapeutic response vary considerably across regions, shaped by differences in parasite species, host characteristics, and environmental conditions (Madusanka et al., 2022; de Vries and Schallig, 2022). Reliable, region-specific data are therefore essential to enhance diagnostic accuracy, guide treatment decisions, and inform control strategies.

Accordingly, the present study aimed to describe the demographic, clinical, and therapeutic characteristics of patients with CL in Mashhad, the second most populous city in Iran and an urban hotspot of CL transmission (Mohammadi et al., 2025). Using nearly a decade of registry-based data from three major medical centers, we specifically aimed to characterize lesion types, anatomical distribution, seasonal and temporal trends, and treatment outcomes among confirmed cases. These data are intended to support evidence-based, region-specific disease management and public health planning.

## 2 Method and material

### 2.1 Study design

This retrospective case-series study was conducted assessed all individuals (*n* = 2031) who referred to the Cutaneous Leishmaniasis Research Center bases in *Imam*-Reza hospital, Ghaem hospital, and Abo-Bargh health center (Mashhad, Iran) due to suspected CL during 2015–24. The research adhered to the STROBE guidelines for reporting observational studies in epidemiology (Cuschieri, 2019).

### 2.2 Study area

Mashhad, the capital of Razavi Khorasan Province in northeastern Iran, occupies about 351 km^2^ of urban area. The city lies at roughly 36°19′N 59°32′E in the northeastern part of the country. Mashhad's climate is classified as cold semi-arid, characterized by hot, dry summers and cool winters.

### 2.3 Study population and data collection

Patients with a clinical suspicion of CL who were referred to the Cutaneous Leishmaniasis Research Center bases across Mashhad city between 2015 and 2024 were consecutively enrolled in the study. After being examined by a physician in the health center, the patients provided informed consents and completed a special questionnaire including demographic informations like age, sex, area of residence, and educational level. Diagnosis of CL was primarily confirmed by direct microscopic examination of skin smears. Skin biopsy or polymerase chain reaction (PCR) testing was performed selectively in cases with atypical clinical presentation, unusual lesion morphology, or diagnostic uncertainty, and was not routinely used for species identification.

**Fig. 1:**
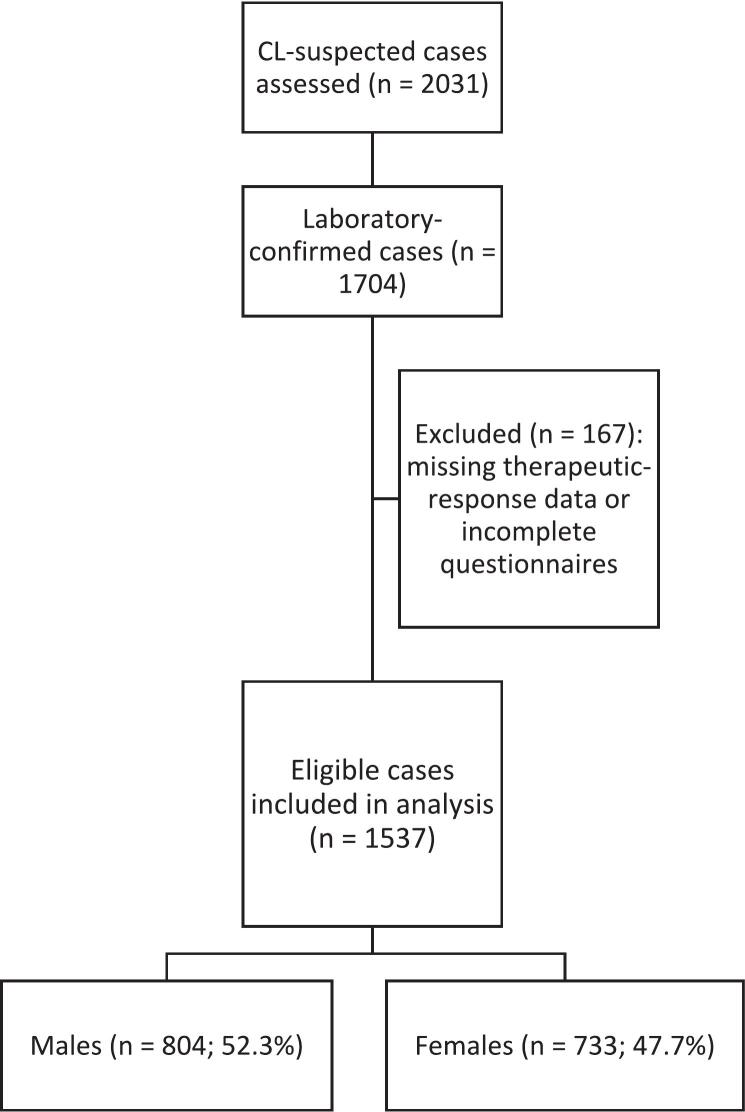
Flow diagram of patient selection for the study.

After confirming the diagnosis of CL through clinical evaluation and parasitological testing, we documented the lesion characteristics, including anatomical location, number of lesions, and lesion type. Following data collection, patients received treatment according to established therapeutic protocols (World Health Organization, 2014). Each patient was subsequently followed up at three-month intervals, during which treatment progression and clinical outcomes were assessed and recorded. Case selection process for patients included in the present study is shown in Fig. 1.

### 2.4 Definition of terms

Cutaneous lesions in this study presented in several morphological forms, including papular, nodular, ulcerative, plaque type, lupoid, and some uncommon forms like sporotrichoid, and erysipeloid types, marked as “others” due to their scarcity. Because healing patterns differ across these presentations, outcome definitions were adapted to each form. For ulcerative lesions, complete clinical cure was defined as full epithelialization of the ulcer base with no induration, crusting, or discharge, and absence of new or secondary lesions. Partial response referred to at least a 50% reduction in lesion area together with visible improvement in inflammation or induration. Therapeutic failure was defined as <50% re-epithelization of the area of the lesion. For papular and nodular lesions, complete clinical cure was defined as complete regression of lesion activity, marked by flattening of the surface, disappearance of induration, and the appearance of residual pigmentation or an atrophic scar without any new lesions. Partial response was defined as a reduction of 50% or more in lesion size or palpable infiltration compared with baseline. Therapeutic failure was defined as <50% reduction of the area of the lesion. For lupoid lesions, which often show a prolonged and relapsing course, complete cure was defined as full regression of infiltrated plaques and nodules with no scaling or active borders, confirmed at two consecutive visits at least three months apart. Patients were evaluated at baseline and followed at approximately 45, 90, and 180 days after treatment initiation. Cure was confirmed only when the relevant criteria were met at both the 90-day and 180-day assessments. Patients previously declared cured of CL by a physician following completion of a full course of systemic or local therapy, who subsequently presented with reactivation of apparently healed lesion(s), were classified as relapse cases. These definitions were derived and slightly adapted from previously published clinical studies and international guidelines on human CL (Aronson et al., 2016; Castro et al., 2017; Solomon et al., 2022).

### 2.5 Statistical analysis

Data analyses were conducted utilizing R version 4.5.1 (R Core Team, 2025). Before conducting the analysis, the data underwent a thorough cleaning process and were evaluated for any missing values and outliers. The quantile-quantile plot was utilized to assess the normality assumptions for continuous variables. Frequencies and percentages are provided for categorical variables in the descriptive statistics. Continuous variables are reported as mean ± standard deviation for data that follows a normal distribution, or as median with interquartile range for data that does not follow a normal distribution.

### 2.6 Ethical statement

This investigation was carried out in alignment with the Declaration of Helsinki and obtained approval from the Ethics Committee of Mashhad University of Medical Sciences, under the approval code IR.MUMS.IRH.REC.1404.052. Each item for agreement was detailed separately to secure written consent from those who chose to participate in the study. Clinical photographs were obtained with written informed consent from the patients (or their guardians) for publication of anonymized images.

## 3 Results

**Fig. 2:**
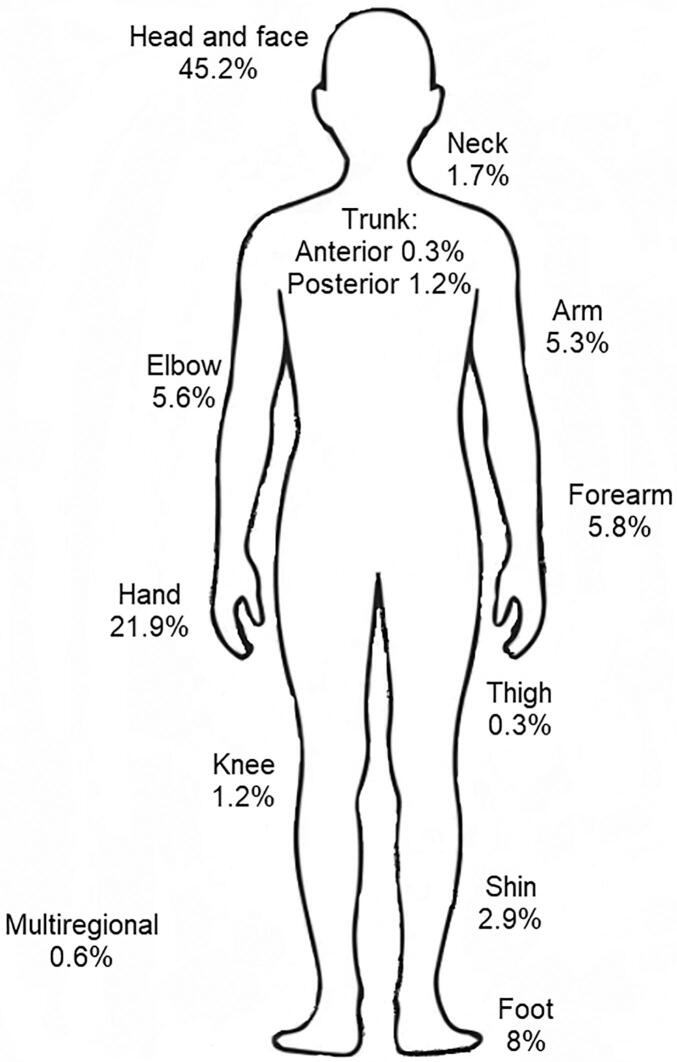
Percentage of cutaneous leishmaniasis lesions recorded at different anatomical sites among examined patients.

**Fig. 3:**
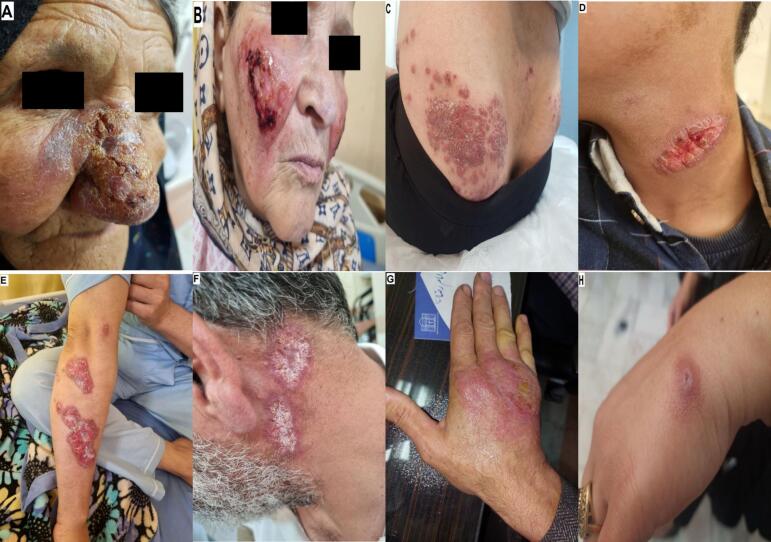
Representative images of cutaneous leishmaniasis lesions in patients, showing variable clinical appearances and anatomical sites. All photographs were taken during clinical visits after obtaining informed consent.

A total of 1537 patients were confirmed with CL during the study period. Adults had a median age of 45 years (IQR 32–57), while children showed a median of 7 years (IQR 3–10.5). Male patients made up 52.3% of all cases. Most patients were of Persian ethnicity (93.8%) and lived in peri-urban areas (59.4%). Education was generally low between patients. These characteristics were similar across the three hospitals involved (Table 1). Localized disease dominated the sample, observed in majority of patients (99.4%). A single lesion was recorded in just over 50% of cases. Lesions appeared mainly on exposed parts of the body - the head and neck in 47.0%, upper limbs in 38.6%, and lower limbs in 12.3%. Multiple-site involvement was rare (0.6%). Fig. 2 shows distribution of CL lesions by anatomic region showing detailed localization beyond the grouped extremity categories. Different examples of the CL clinical presentations are shown in Fig. 3.

**Table 1:** Demographic and clinical characteristics of patients with confirmed cutaneous leishmaniasis.

Variable	Total, *n* = 1537 (%)	*Imam* Reza, *n* = 438 (28.5%)	Ghaem, *n* = 672 (43.7%)	Ab-o Bargh, *n* = 427 (27.8%)
Demographic information				
Age, median (IQR)				
Adults	45.00 [32.00–57.00]	44.00 [33.00–58.00]	45.00 [32.00–57.00]	45.00 [31.00–57.00]
Children	7.00 [3.00–10.50]	7.00 [3.00–10.00]	7.00 [3.00–10.00]	8.00 [3.00–11.00]
Sex, n (%)				
Male	804 (52.3%)	234 (53.4%)	346 (51.5%)	224 (52.5%)
Female	733 (47.7%)	204 (46.6%)	326 (48.5%)	203 (47.5%)
Ethnicity, n (%)				
Persian	1442 (93.8%)	380 (86.8%)	650 (96.7%)	412 (96.5%)
Afghan	66 (4.3%)	29 (6.6%)	22 (3.3%)	15 (3.5%)
Turkmen	12 (0.8%)	0 (0.0%)	12 (2.7%)	0 (0.0%)
Arab	4 (0.3%)	4 (0.9%)	0 (0.0%)	0 (0.0%)
Kurd	5 (0.3%)	0 (0.0%)	0 (0.0%)	5 (1.1%)
Turk	8 (0.5%)	8 (1.8%)	0 (0.0%)	0 (0.0%)
Educational level, n (%)				
Low (below high school)	1101 (71.6%)	319 (72.8%)	487 (72.5%)	295 (69.1%)
Medium (high school or equivalent)	258 (16.8%)	70 (16.0%)	109 (16.2%)	79 (18.5%)
High (above high school)	178 (11.6%)	49 (11.2%)	76 (11.3%)	53 (12.4%)
Marriage status, n (%)				
Single	844 (54.9%)	257 (58.7%)	361 (53.7%)	226 (52.9%)
Married	674 (43.9%)	175 (40.0%)	304 (45.2%)	195 (45.7%)
Divorced	19 (1.2%)	6 (1.4%)	7 (1.0%)	6 (1.4%)
Area of residence, n (%)				
Urban	624 (40.6%)	208 (47.5%)	146 (21.7%)	270 (63.2%)
Peri-urban	913 (59.4%)	230 (52.5%)	526 (78.3%)	157 (36.8%)
Past medical history				
Immunodeficiency, n (%)	8 (0.5%)	4 (0.9%)	2 (0.3%)	2 (0.5%)
Cardiovascular disease, n (%)	77 (5.0%)	22 (5.0%)	34 (5.1%)	21 (4.9%)
Hepatic or Gastrointestinal, n (%)	9 (0.6%)	3 (0.7%)	3 (0.4%)	3 (0.7%)
Renal disease, n (%)	5 (0.3%)	1 (0.2%)	3 (0.4%)	1 (0.2%)
Diabetes (Type 1 or 2), n (%)	50 (3.3%)	13 (3.0%)	25 (3.7%)	12 (2.8%)
Dermatologic disease, n (%)	9 (0.6%)	3 (0.7%)	3 (0.4%)	3 (0.7%)
None, n (%)	1379 (89.7%)	392 (89.5%)	602 (89.6%)	385 (90.2%)
Characteristics of cutaneous lesions				
Form of the disease, n (%)				
Localized	1528 (99.4%)	435 (99.3%)	666 (99.1%)	427 (100.0%)
Diffuse	9 (0.6%)	3 (0.7%)	6 (0.9%)	0 (0.0%)
Number of lesions, n (%)				
1	816 (53.1%)	235 (53.7%)	348 (51.8%)	233 (54.6%)
2	357 (23.2%)	89 (20.3%)	179 (26.6%)	89 (20.8%)
3	126 (8.2%)	36 (8.2%)	54 (8.0%)	36 (8.4%)
≥4	238 (15.5%)	78 (17.8%)	91 (13.5%)	69 (16.2%)
Anatomic site, n (%)				
Head and neck	723 (47.0%)	204 (46.6%)	319 (47.5%)	200 (46.8%)
Upper extremities	593 (38.6%)	172 (39.3%)	251 (37.4%)	170 (39.8%)
Lower extremities	189 (12.3%)	53 (12.1%)	85 (12.6%)	51 (11.9%)
Trunk	23 (1.5%)	6 (1.4%)	11 (1.6%)	6 (1.4%)
Multiple sites	9 (0.6%)	3 (0.7%)	6 (0.9%)	0 (0.0%)
Type of lesions, n (%)				
Populonodular	775 (50.4%)	56 (12.8%)	435 (64.7%)	284 (66.5%)
Plaque	210 (13.7%)	58 (13.2%)	93 (13.8%)	59 (13.8%)
Ulcerative	476 (31.0%)	284 (64.8%)	118 (17.6%)	74 (17.3%)
Lupoid	68 (4.4%)	32 (7.3%)	26 (3.9%)	10 (2.3%)
Others	8 (0.5%)	8 (1.8%)	0 (0.0%)	0 (0.0%)
Treatment				
Parenteral antimonials, n (%)	122 (7.9%)	122 (27.9%)	0 (0.0%)	0 (0.0%)
Intralesional antimonials, n (%)	911 (59.3%)	261 (59.6%)	429 (63.8%)	221 (51.8%)
Miltefosine, n (%)	58 (3.8%)	13 (3.0%)	28 (4.2%)	17 (4.0%)
Intralesional Liposomal Amphotericin B, n (%)	30 (2.0%)	0 (0.0%)	23 (3.4%)	7 (1.6%)
Parenteral Liposomal Amphotericin B, n (%)	114 (7.4%)	10 (2.3%)	56 (8.3%)	48 (11.2%)
Cryotherapy, n (%)	197 (12.8%)	17 (3.9%)	71 (10.6%)	109 (25.5%)
Electrothermotherapy, n (%)	7 (0.5%)	0 (0.0%)	7 (1.0%)	0 (0.0%)
Itraconazole, n (%)	72 (4.7%)	15 (3.4%)	32 (4.8%)	25 (5.9%)
Azithromycin, n (%)	26 (1.7%)	0 (0.0%)	26 (3.9%)	0 (0.0%)
Outcome				
Complete clinical cure, n (%)	964 (62.7%)	292 (66.7%)	453 (67.4%)	219 (51.3%)
Partial response, n (%)	366 (23.8%)	89 (20.3%)	115 (17.1%)	162 (37.9%)
Therapeutic failure n (%)	133 (8.7%)	56 (12.8%)	31 (4.6%)	46 (10.8%)
Relapse, n (%)	74 (4.8%)	1 (0.2%)	73 (10.9%)	0 (0.0%)

**Fig. 4:**
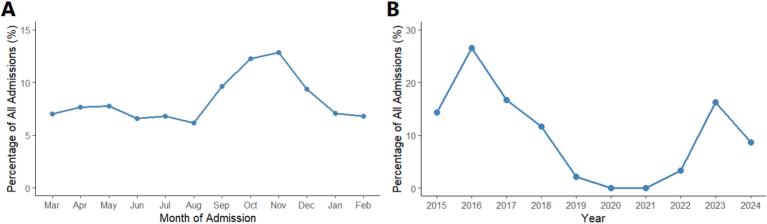
Temporal distribution of cutaneous leishmaniasis cases by month (A) and year (B).

The papulonodular form was most common (50.4%), while ulcerative (31.0%), plaque form (13.7%), and lupoid (4.4%) lesions followed. Children younger than 10 years of age, represented roughly one-third of the cases (Table 2). The share of male patients was higher among adolescents and younger adults but decreased with age. In older groups, women accounted for a greater proportion. Disease occurrence dropped gradually after the fifth decade of life, yet a small number of elderly cases persisted. Case numbers varied from year to year without a fixed direction (Table 3). Most diagnoses occurred from October to December, with a noticeable dip during spring and early summer (Figs. 4A). The number of patients markedly declined during the latter months of 2019, 2020, and 2021, largely due to the impact of the Coronavirus Disease 2019 (COVID-19) pandemic and the associated public-health restrictions implemented during that period. Following the pandemic, as the COVID-19 restrictions were lifted, the number of patients increased again in 2022 and 2023 (Fig. 4B).

**Table 2:** Age-specific distribution of patients with cutaneous leishmaniasis by gender.

Variable	Total, n (%)	Male, n (%)	Female, n (%)
Age (years)			
0–10, n (%)	536 (34.9%)	248 (46.3%)	288 (53.7%)
11–20, n (%)	196 (12.8%)	120 (61.2%)	76 (38.8%)
21–30, n (%)	157 (10.2%)	88 (56.1%)	69 (43.9%)
31–40, n (%)	161 (10.5%)	98 (60.9%)	63 (39.1%)
41–50, n (%)	168 (10.9%)	98 (58.3%)	70 (41.7%)
51–60, n (%)	187 (12.2%)	98 (52.4%)	89 (47.6%)
61–70, n (%)	85 (5.5%)	30 (35.3%)	55 (64.7%)
71–80, n (%)	28 (1.8%)	16 (57.1%)	12 (42.9%)
80–90, n (%)	15 (1.0%)	6 (40.0%)	9 (60.0%)
91–100, n (%)	4 (0.3%)	2 (50.0%)	2 (50.0%)
Total, n (%)	1537 (100.0%)	804 (52.3%)	733 (47.7%)

**Table 3:** Annual and monthly distribution of cutaneous leishmaniasis cases by gender (F: Female, M; Male) and year of examination.

Month of examination	Gender	Year of examination											
		Jan, n (%)	Feb, n (%)	Mar, n (%)	Apr, n (%)	May, n (%)	Jun, n (%)	Jul, n (%)	Aug, n (%)	Sep, n (%)	Oct, n (%)	Nov, n (%)	Dec, n (%)
2015, n (%)	F	7 (6.8%)	1 (1.0%)	11 (10.7%)	7 (6.8%)	7 (6.8%)	9 (8.7%)	9 (8.7%)	5 (4.9%)	12 (11.7%)	8 (7.8%)	13 (12.6%)	14 (13.6%)
	M	1 (0.8%)	1 (0.8%)	12 (10.2%)	14 (11.9%)	14 (11.9%)	3 (2.5%)	2 (1.7%)	6 (5.1%)	16 (13.6%)	18 (15.3%)	19 (16.1%)	12 (10.2%)
2016, n (%)	F	15 (7.1%)	19 (9.0%)	12 (5.7%)	16 (7.5%)	19 (9.0%)	11 (5.2%)	7 (3.3%)	19 (9.0%)	16 (7.5%)	19 (9.0%)	41 (19.3%)	18 (8.5%)
	M	15 (7.7%)	15 (7.7%)	18 (9.2%)	20 (10.2%)	22 (11.2%)	11 (5.6%)	4 (2.0%)	12 (6.1%)	16 (8.2%)	19 (9.7%)	31 (15.8%)	13 (6.6%)
2017, n (%)	F	7 (6.7%)	10 (9.5%)	1 (1.0%)	0 (0.0%)	0 (0.0%)	13 (12.4%)	17 (16.2%)	10 (9.5%)	12 (11.4%)	18 (17.1%)	7 (6.7%)	10 (9.5%)
	M	14 (9.2%)	10 (6.6%)	9 (5.9%)	0 (0.0%)	2 (1.3%)	6 (3.9%)	24 (15.8%)	18 (11.8%)	27 (17.8%)	17 (11.2%)	13 (8.6%)	12 (7.9%)
2018, n (%)	F	9 (10.5%)	12 (14.0%)	3 (3.5%)	5 (5.8%)	5 (5.8%)	9 (10.5%)	8 (9.3%)	4 (4.7%)	1 (1.2%)	6 (7.0%)	8 (9.3%)	16 (18.6%)
	M	12 (12.8%)	11 (11.7%)	6 (6.4%)	5 (5.3%)	10 (10.6%)	5 (5.3%)	7 (7.4%)	3 (3.2%)	12 (12.8%)	4 (4.3%)	6 (6.4%)	13 (13.8%)
2019, n (%)	F	0 (0.0%)	0 (0.0%)	10 (43.5%)	8 (34.8%)	5 (21.7%)	0 (0.0%)	0 (0.0%)	0 (0.0%)	0 (0.0%)	0 (0.0%)	0 (0.0%)	0 (0.0%)
	M	0 (0.0%)	0 (0.0%)	4 (40.0%)	4 (40.0%)	2 (20.0%)	0 (0.0%)	0 (0.0%)	0 (0.0%)	0 (0.0%)	0 (0.0%)	0 (0.0%)	0 (0.0%)
2020, n (%)	F	0 (0.0%)	0 (0.0%)	0 (0.0%)	0 (0.0%)	0 (0.0%)	0 (0.0%)	0 (0.0%)	0 (0.0%)	0 (0.0%)	0 (0.0%)	0 (0.0%)	0 (0.0%)
	M	0 (0.0%)	0 (0.0%)	0 (0.0%)	0 (0.0%)	1 (100.0%)	0 (0.0%)	0 (0.0%)	0 (0.0%)	0 (0.0%)	0 (0.0%)	0 (0.0%)	0 (0.0%)
2021, n (%)	F	0 (0.0%)	0 (0.0%)	0 (0.0%)	0 (0.0%)	0 (0.0%)	0 (0.0%)	0 (0.0%)	0 (0.0%)	0 (0.0%)	0 (0.0%)	0 (0.0%)	0 (0.0%)
	M	0 (0.0%)	0 (0.0%)	0 (0.0%)	0 (0.0%)	0 (0.0%)	0 (0.0%)	0 (0.0%)	0 (0.0%)	0 (0.0%)	0 (0.0%)	1 (100.0%)	0 (0.0%)
2022, n (%)	F	2 (8.3%)	6 (25.0%)	0 (0.0%)	0 (0.0%)	0 (0.0%)	0 (0.0%)	0 (0.0%)	0 (0.0%)	2 (8.3%)	10 (41.7%)	4 (16.7%)	0 (0.0%)
	M	3 (10.7%)	3 (10.7%)	0 (0.0%)	0 (0.0%)	0 (0.0%)	0 (0.0%)	0 (0.0%)	0 (0.0%)	3 (10.7%)	14 (50.0%)	4 (14.3%)	1 (3.6%)
2023, n (%)	F	9 (8.1%)	5 (4.5%)	7 (6.3%)	18 (16.2%)	9 (8.1%)	11 (9.9%)	6 (5.4%)	2 (1.8%)	6 (5.4%)	12 (10.8%)	12 (10.8%)	14 (12.6%)
	M	6 (4.3%)	12 (8.6%)	11 (7.9%)	11 (7.9%)	14 (10.1%)	14 (10.1%)	10 (7.2%)	7 (5.0%)	7 (5.0%)	14 (10.1%)	19 (13.7%)	14 (10.1%)
2024, n (%)	F	4 (5.8%)	0 (0.0%)	3 (4.3%)	6 (8.7%)	2 (2.9%)	2 (2.9%)	2 (2.9%)	6 (8.7%)	9 (13.0%)	17 (24.6%)	11 (15.9%)	7 (10.1%)
	M	5 (7.7%)	0 (0.0%)	1 (1.5%)	4 (6.2%)	7 (10.8%)	7 (10.8%)	9 (13.8%)	3 (4.6%)	9 (13.8%)	12 (18.5%)	8 (12.3%)	0 (0.0%)
Total, n (%)	F	53 (7.2%)	53 (7.2%)	47 (6.4%)	60 (8.2%)	47 (6.4%)	55 (7.5%)	49 (6.7%)	46 (6.3%)	58 (7.9%)	90 (12.3%)	96 (13.1%)	79 (10.8%)
	M	56 (7.0%)	52 (6.5%)	61 (7.6%)	58 (7.2%)	72 (9.0%)	46 (5.7%)	56 (7.0%)	49 (6.1%)	90 (11.2%)	98 (12.2%)	101 (12.6%)	65 (8.1%)

Intralesional antimonials were the main approach, prescribed for 59.3% of patients. Systemic antimonials were used in 7.9%, and cryotherapy in 12.8%. Other regimens, including miltefosine (3.8%), intralesional liposomal amphotericin B (2.0%), parenteral liposomal amphotericin B (7.4%), itraconazole (4.7%), and azithromycin (1.7%), were less frequent. The choice of therapy often reflected lesion characteristics and physician preference rather than a standardized protocol.

Outcomes varied across regimens. Complete clinical cure was achieved in 62.7% of all patients. Partial response accounted for 23.8%, treatment failure for 8.7%, and relapse for 4.8%. The highest cure proportion appeared in systemic antimonial therapy (87.7%), followed by intralesional antimonials (63.7%) and parenteral liposomal amphotericin B (62.3%). Intralesional liposomal amphotericin B showed modest success (43.3%), and azithromycin performed poorest (38.5%). Relapse occurred most often after treatment with azithromycin (Table 4).

**Table 4:** Outcomes according to anti-leishmaniasis regimen in patients with confirmed cutaneous leishmaniasis.

Outcome	Parenteral antimonials, n (%)	Intralesional antimonials, n (%)	Miltefosine, n (%)	Intralesional Liposomal Amphotericin B, n (%)	Parenteral Liposomal Amphotericin B, n (%)	Cryotherapy, n (%)	Electrothermotherapy, n (%)	Itraconazole, n (%)	Azithromycin, n (%)	Total, n (%)
Complete clinical cure, n (%)	107 (87.7%)	580 (63.7%)	39 (67.2%)	13 (43.3%)	71 (62.3%)	97 (49.2%)	2 (28.6%)	45 (62.5%)	10 (38.5%)	964 (62.7%)
Partial response, n (%)	2 (1.6%)	207 (22.7%)	18 (31.0%)	12 (40.0%)	33 (28.9%)	71 (36.0%)	0 (0.0%)	16 (22.2%)	7 (26.9%)	366 (23.8%)
Therapeutic failure, n (%)	13 (10.7%)	74 (8.1%)	1 (1.7%)	0 (0.0%)	4 (3.5%)	28 (14.2%)	5 (71.4%)	7 (9.7%)	1 (3.8%)	133 (8.7%)
Relapse, n (%)	0 (0.0%)	50 (5.5%)	0 (0.0%)	5 (16.7%)	6 (5.3%)	1 (0.5%)	0 (0.0%)	4 (5.6%)	8 (30.8%)	74 (4.8%)

**Fig. 5:**
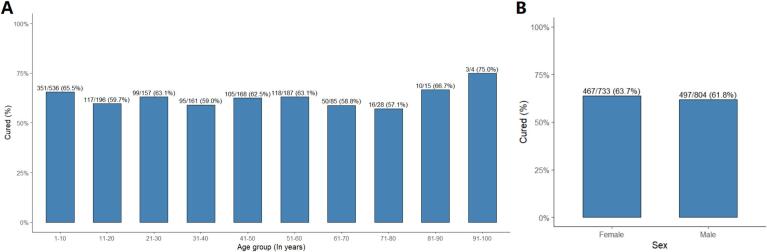
Proportion of patients achieving complete clinical cure by lesion type in cutaneous leishmaniasis by (A) age group, (B) gender.

Fig. 5A shows the treatment outcome by age group. The overall cure rate remained relatively stable across age categories, ranging from about 59% to 66% in most groups. The highest rate of cure was observed among individuals aged 91–100 years (75.0%), while the lowest occurred in the 61–70 year-old age group (58.8%). Fig. 5B compares cure rates between genders. The proportion of cured cases was slightly higher among females (63.7%) than males (61.8%).

## 4 Discussion

In this large retrospective case series of 1537 patients with CL from Mashhad, northeastern Iran, we observed that children and more specifically children with the age of less than 10 years consisted the greatest proportion of patients in this study. Male participants were slightly higher than female counterparts. Education level was among the patients was generally low and mostly, patients were residents of peri-urban areas. The lesions were mostly distributed in exposed areas such as head and neck followed by upper extremities. The most prevalent clinical form was populonodular followed by ulcerative and plaque-type lesion. The most frequent treatment option was intralesional antimony, and nearly 63% of all patient who underwent different treatment options were completely cured. CL showed a peak in autumn and slowly diminished in winter, with another surge in spring. Patient attendance showed a notable drop toward the end of 2019, 2020, and 2021, which coincided with the COVID-19 pandemic and the enforcement of control measures limiting healthcare access.

Our finding that most CL patients were young (especially <10 years) and slightly more often male echoes patterns seen elsewhere. For example, a recent Iranian nationwide analysis found that the largest age-group of CL cases was children under 10 (23%) (Sharifi et al., 2023). Likewise, a regional Iranian study reported that the 0–14 year age group had the highest CL incidence (Khazaei et al., n.d.). However, in contrast some other works reported that CL was more prevalent in older patients. In a study by Rajabi-Gharaii et al. (Rajabi Gharaii et al., 2025) they reported that 41% of the patients with CL were middle-aged. In another study from Brazil (Murback et al., 2011), and Saudi arabia (Al-Dhafiri et al., 2023), also CL patients were more from adult age groups. These differences in age distribution likely reflect the epidemiological characteristics of each transmission setting. In long-standing or hyperendemic areas, repeated exposure over time leads to partial immunity and reduced susceptibility among adults, while children, who lack previously acquired immunity, constitute the majority of symptomatic cases (Reithinger et al., 2007). Conversely, regions reporting higher incidence in adults may represent emerging foci or contexts in which occupational, behavioural, or ecological factors shift exposure toward older age groups (Reithinger et al., 2007).

According to our results, among the patients included in the present study, male participants constructed a greater proportion of our cohort. This male predominance in CL infection is consistent with findings from previous studies. For instance, a study from the western region of Saudi Arabia reported a similar pattern, with 406 male and only 61 female patients among a total of 467 cases (Elmekki et al., 2017). Comparable gender disparities have been observed elsewhere: in Ethiopia (Bisetegn et al., 2020); in Yemen (Asmaa et al., 2017); in Libya (Amro et al., 2012); and in Iraq (Al-Khayat et al., 2018). A plausible explanation for this gender difference is that women's traditional practice of wearing the Hijab (Islamic attire) in these countries provides partial protection against sand fly bites, whereas men's greater involvement in outdoor and nighttime activities increases their exposure risk (Sarmadi et al., 2023). Interestingly, a similar male predominance has also been reported in studies from Europe, despite the absence of such traditional clothing practices (Guery et al., 2021; Glans et al., 2018). However, a substantial proportion of patients in these European cohorts were migrants or travelers originating from endemic MENA regions, and information on their clothing habits or exposure conditions during residence in Europe was not available. Therefore, the extent to which protective clothing contributed to the observed gender pattern in these populations remains uncertain.

In agreement with the literature, most of our lesions occurred on exposed skin. In a study from central Iran, roughly 80% of lesion distribution were on hands or feet (Nateghi Rostami et al., 2013). In a study from central America, in children, CL lesions were more distributed on facial area. In contrast among older patients, the lesions were more distributed on extrimities, in favor of lower extrimities (Castro et al., 2023). In another study from Sri Lanka, the smear positive (established cases) patients were found to have higher distribution of lesions over their head and neck (Iddawela et al., 2018), which was aligned with our findings. Among the patients studied, 53.1% had a single lesion, 23.2% had two lesions, 8.2% had three lesions, and 15.5% presented with four or more than four lesions. The predominance of single-lesion cases is consistent with previous reports from Iraq (49.3%) (Al-Khayat et al., 2018), Saudi Arabia (38.4%) (Al-Dhafiri et al., 2023), Sri Lanka (76%) (Galgamuwa et al., 2017), and Iran (61.3%) (Ahmadi et al., 2013), where the majority of patients similarly exhibited only one lesion.

For the clinical form of the lesions, we observed mostly papulonodular lesions. In the study by Iddawela and colleagues (Iddawela et al., 2018), papulonodular form was more prevalent between smear positive cases (46%). In another study by Karami et al. (Karami et al., 2013), nodular lesions were more frequent among participants (30.4%), closely followed by ulcerative form (29.3%).

Discussing the monthly and seasonal distribution, we observed that disease incidence increases as temperatures drop, starting in early autumn. CL incidence typically rises in late summer/early autumn and falls through winter. A national time-series found cases “gradually declined from spring to July, then sharply upsurged August-November and dropped again by March” (Sharifi et al., 2023). Our data parallels these findings and matches the pattern seen in Isfahan, where 54% of cases occurred in fall (Karami et al., 2013). Yearly trend showed us a drop in CL cases during 2019–2021, likely due to COVID-19-related disruptions. Studies in Iran reported that CL incidence declined after the onset of the COVID-19 pandemic, likely due to lockdowns and healthcare disruptions. For instance, Shams et al. found a decreasing incidence of CL in Ilam province in western Iran during the COVID-19 pandemic (2020−2021), possibly because of the disruption of CL diagnosis and treatment follow-up (Shams et al., 2023). These effects were also seen in Al-Ahsa, where cases dipped sharply in 2020–2021 before resurging in 2022 and 2023, possibly due to resumed activity and diagnostic services (Al-Dhafiri et al., 2023). Moreover, another plausible explanation is that as people had to stay indoors, they were less exposed to sandflies, which could lead to fewer number of cases (Sabetkish and Rahmani, 2021). However, this trend was not consistent across all endemic regions. As discussed by Shams et al. (Shams et al., 2023), in some areas the number of CL cases remained stable or even increased, largely due to the diversion of health resources and funding toward COVID-19 control measures. The pandemic caused a temporary suspension of key anti-leishmaniasis activities, such as rodent control, insecticide spraying, and environmental vector management, as health personnel and budgets were redirected to COVID-19 prevention and treatment.

In our cohort, most patients were treated with antimonials in either systemic or intralesional form, according to lesion type and clinical indication. Systemic administration of antimonials achieved the highest cure rate (87.7%), a value notably higher than those reported in broader meta-analyses. Tuon et al. (Tuon et al., 2008) reviewed over 1100 patients with New World CL and observed a pooled efficacy of 76.5% for pentavalent antimonials. Their analysis focused on New World species, primarily *L. braziliensis* and *L. panamensis*. It is worth noting that the species circulating in our region, including L. *major* and L. *tropica*, belong to the Old World group (Karimian Shirazi et al., 2014; Mahmoodi et al., 2010). Nonetheless, it is worth noting that our study represents Old World species, mainly L. *major* and *L. tropica*, while the meta-analysis by Tuon et al. focused on New World species such as L. *braziliensis* and L. *panamensis*. This distinction limits the direct comparability of cure rates and highlights the need for region-specific evaluations of treatment performance.

In a European multicenter study by Glans et al. (Glans et al., 2022) on 206 cases, pentavalent antimony yielded cure rates of 73% for L. *major* and 78% for L. *tropica*, with intralesional therapy reaching 86% and systemic therapy 67%. They reported overall cure rates of 54.6% for systemic antimonials in children and 68.2% in older adults. Our findings exceed both values, suggesting better outcomes in the general adult population. Another study by Castro et al. (Castro et al., 2023) showed that miltefosine has an overall cure rate of nearly 56% for both children and adults. In our study we observed 67.2%. results from other studies reported cure rates from 48% to 77% (van Henten et al., 2021; Ware et al., 2020). This heterogeneity in existing results warrants further studies to achieve more reliable estimates of efficacy across different endemic settings.

For the treatment of CL with intralesional Amphotericin B the data is limited in comparison to other treatment strategies. A clinical trial reported that 61.4% of the patients who were involved in the study were recovered completely (more than 90% reduction in size and induration), 21.6% had partial remission (Goyonlo et al., 2014). We observed 43.3% and 40.0% cure rate and partial remission rate, respectively. Notably, another clinical trial demonstrated that intralesional amphotericin B performs comparably to intralesional antimonials, suggesting that despite the sparse literature, its therapeutic potential is at least on par with established local treatments (Layegh et al., 2011). For the systemic administration of Amphotericin B, we observed 62.3% cure rate in treatment of CL. Published data show that systemic liposomal amphotericin B achieves cure rates of about 55–90% in CL (Machado et al., 2015; Barroso et al., 2022; Chivinski et al., 2023).

Cure rates of 62.5% for itraconazole and 38.5% for azithromycin were observed in our study. The efficacy of itraconazole aligns with prior literature, which generally reports around 60–65%, particularly in cases involving L. *major*, while its performance against L. *tropica* is substantially lower, with pooled estimates closer to 15% (Galvão et al., 2017). For azithromycin, while favorable cure rates have been reported in cases of L. *braziliensis* infection in New World CL (Prata et al., 2003), its effectiveness against Old World species has been unsatisfactory (Momeni et al., 2006; Layegh et al., 2007).

Cryotherapy and electrothermotherapy were not as effective as reported in other studies (Madusanka et al., 2022), probably due to differences in lesion characteristics, treatment application techniques, and follow-up adherence, as well as variations in the endemic parasite species. Overall, the observed variability in treatment outcomes in this cohort likely reflects a combination of host immune response, lesion chronicity, treatment adherence, and real-world clinical practice rather than differences attributable to a single therapeutic agent (Bamorovat et al., 2023). Chronic or long-standing lesions, which are particularly lupoid or recurrent forms, may be associated with altered local immune responses and reduced responsiveness to standard therapies, thereby increasing the risk of partial response or relapse (Bamorovat et al., 2021). In addition, adherence to treatment regimens, especially those requiring repeated intralesional injections or prolonged systemic administration, may be influenced by patient age, socioeconomic status, and access to follow-up care, which can affect observed effectiveness in routine clinical settings (Bamorovat et al., 2023; Bamorovat et al., 2021; Aflatoonian et al., 2022). Given the coexistence of zoonotic and anthroponotic transmission cycles in Mashhad, heterogeneity in clinical response is expected, and treatment outcomes should therefore be interpreted at the population level rather than as species- or drug-specific efficacy estimates.

This study provides the most comprehensive, decade-long registry analysis of CL in Mashhad, an urban hyperendemic focus where long-term clinical data have been scarce. By integrating detailed demographic, clinical, and treatment information from more than 1500 confirmed cases, it fills a critical gap in understanding age-specific vulnerability, lesion morphology, and real-world therapeutic performance in northeastern Iran. Unlike previous fragmented or short-term reports, our dataset allows characterization of seasonal trends, COVID-19-related disruptions in case detection, and the full clinical spectrum of CL, including uncommon morphological variants. Importantly, we present comparative effectiveness data for commonly used treatments in Old World CL, which is not that extensively studied in the literature and is essential for guiding local clinical decision-making. Together, these findings provide an updated, region-specific evidence base that can support surveillance, case management, and future research in one of Iran's most affected urban settings. Nevertheless, this study had some limitations. This study was based on passive surveillance data derived from patients who presented to referral hospitals and health centers. As such, the findings reflect health-seeking behavior and may underestimate the true burden of CL in the community, particularly among individuals with mild, self-healing lesions or limited access to healthcare services. Active case-finding strategies were not employed, and therefore asymptomatic or untreated cases were not captured. These factors should be considered when interpreting incidence patterns and generalizing the findings to the broader population. Being retrospective, it relied on recorded data and therefore may have been affected by incomplete documentation or variable follow-up intervals. Although the diagnostic approach in our center was standardized and relied primarily on microscopic confirmation, molecular species identification was not routinely performed for individual cases. This limits the ability to attribute specific clinical manifestations or treatment outcomes to individual *Leishmania* species. Nevertheless, multiple molecular epidemiological studies conducted in Mashhad and northeastern Iran have consistently demonstrated that *Leishmania tropica* (anthroponotic cutaneous leishmaniasis) and *Leishmania major* (zoonotic cutaneous leishmaniasis) are the predominant circulating species in this region (Karimian Shirazi et al., 2014; Mahmoodi et al., 2010). Accordingly, while species-specific analyses were not feasible in the present registry-based study, the observed clinical and therapeutic patterns likely reflect the combined epidemiology of these two well-established endemic species and should be interpreted at the population level.

Treatment allocation was not randomized but guided by clinical judgment, so unmeasured confounding by lesion severity or patient characteristics is possible. Despite these constraints, the large sample size, standardized follow-up, and decade-long observation period strengthen the reliability of the findings.

## 5 Conclusion

According to our findings, CL remains a significant public health concern in Mashhad, as the second most populous city in Iran. Despite the number of documented cases, the true burden is likely underestimated, underscoring the need for more accurate surveillance and research. Our results also highlight the influence of demographic and climatic factors. Although the overall cure rate observed in our study was encouraging, it should be interpreted with caution, as the data were derived from observational records rather than controlled clinical trials. To better understand treatment effectiveness and disease progression, future longitudinal studies and randomized clinical trials are warranted. Strengthening public awareness, promoting intersectoral cooperation between hospitals and community health centers, and ensuring timely health communication, particularly in endemic and hyperendemic areas, remain essential components of effective leishmaniasis control and prevention strategies.

## CRediT authorship contribution statement

**Vahid Mashayekhi-Goyonlo:** Writing – review & editing, Supervision, Project administration, Methodology, Conceptualization. **Pouran Layegh:** Writing – review & editing, Supervision, Project administration, Conceptualization. **Zahra Ghasemi:** Writing – review & editing, Writing – original draft, Methodology, Data curation. **Masomeh Hosseini-Nezhad:** Writing – original draft, Methodology, Data curation. **Ali Tajik:** Writing – review & editing, Writing – original draft, Supervision, Project administration, Methodology, Formal analysis, Data curation, Conceptualization.

## Informed consent statement

Informed consent was obtained from all subjects or their guardians, who participated in this study.

## Institutional review board statement

This study adhered to the principles of the Declaration of Helsinki and received ethical clearance from the Ethics Committee of Mashhad University of Medical Sciences (approval code: IR.MUMS.IRH.REC.1404.052).

## Funding

This research was financially supported by 10.13039/501100004748Mashhad University of Medical Sciences (Grant No. 4040124).

## Declaration of competing interest

The authors declare that they have no known competing financial interests or personal relationships that could have appeared to influence the work reported in this paper.

## Data Availability

The anonymized data might be available from the corresponding author upon reasonable request.
